# Active collaboration with primary care providers increases specialist referral in chronic renal disease

**DOI:** 10.1186/1471-2369-5-16

**Published:** 2004-10-22

**Authors:** Adrian Mondry, Ai-Ling Zhu, Marie Loh, Thuy D Vo, Kai Hahn

**Affiliations:** 1 Bioinformatics Institute, Singapore, Republic of Singapore; 2 Klinikum Solingen, Solingen, Germany; 3 Dialysegemeinschaftspraxis Karl- Harr- Strasse, Dortmund, Germany

## Abstract

**Background:**

Late referral to specialist nephrological care is associated with increased morbidity, mortality, and cost. Consequently, nephrologists' associations recommend early referral. The recommendations' effectiveness remains questionable: 22–51% of referrals need renal replacement therapy (RRT) within 3–4 months. This may be due to these recommendations addressing the specialist, rather than the primary care providers (PCP).

The potential of specialist intervention aiming at slowing progression of chronic renal failure was introduced individually to some 250 local PCPs, and referral strategies were discussed. To overcome the PCPs' most often expressed fears, every referred patient was asked to report back to his PCP immediately after the initial specialist examination, and new medications were prescribed directly, and thus allotted to the nephrologist's budget.

**Methods:**

In retrospective analysis, the stage of renal disease in patients referred within three months before the introductory round (group A, n = 18), was compared to referrals two years later (group B, n = 50).

**Results:**

Relative number of patients remained stable (28%) for mild/ moderate chronic kidney disease (MMCKD), while there was a noticeable shift from patients referred severe chronic kidney disease (SCKD) (group A: 44%, group B: 20%) to patients referred in moderate chronic kidney disease (MCKD) (group A: 28%, group B: 52%).

**Conclusion:**

Individually addressing PCPs' ignorance and concerns noticeably decreased late referral. This stresses the importance of enhancing the PCPs' problem awareness and knowledge of available resources in order to ensure timely specialist referral.

## Background

Late referral to specialist care for renal failure is associated with increased morbidity, mortality, and cost (review in[1,2]). Consequently, nephrologists' associations recommend early referral[3,4]. The recently published ERA/ EDTA guideline states: "Referral to nephrology should be considered when the GFR is <60 ml/min and is mandatory when the GFR is <30 ml/min"[3]. The recommendations' effectiveness remains questionable: 22–51% of patients need renal replacement therapy (RRT) within 3–4 months [5,6] after first referral to specialist nephrological care.

Chronic renal disease may be asymptomatic for a very long time. This stresses the importance of primary care providers in ensuring timely referral to a renal care center. Levin[1] reviewed aspects of the referral process and noted that the consequences of late referral were usually and most specifically described in specialist nephrological journals, thus not reaching the necessary target audience, the primary care providers (PCPs).

The problem of late referral seems to be ubiquitous, if discussions with colleagues world- wide may be believed, and has led to numerous local, regional and national initiatives aiming at ensuring timely referral into specialist care. Some initiatives[7,8] successfully bypass the PCPs by introducing population screening programs, while others opt for the alternative of "managed care"[9].

This article summarizes findings from a local initiative started by one of the authors (K.H.) in 1997 in the city of Dortmund, Germany.

In Germany, about 90% of the population are covered by the mandatory health insurance system ("Gesetzliche Krankenversicherung"). All physicians who wish to treat these patients are organized in associations, which negotiate a budget with the insurance companies. Depending on the specialty, a typical per capita budget is then assigned per patient for a period of three months. This budget covers consultation fees, and the cost of medication prescribed. If a physicians exceeds the total budget thus calculated for his practice, the insurance companies can demand restoration of funds. Typically, a GP's per patient budget would be much lower than a nephrologist's. During the time period described here, the nephrologist's budget per patient was about ten times as much than a general practitioner's. While this budget strategy is intended as a safeguard against excess prescriptions, it has been criticized by many physicians for inducing sub- optimal treatment as practice owners comply rather with budget demands than with best practice guidelines.

Dortmund, a town of 589000, has a physician to patient ratio of 141/ 100000 (German average: 156/ 100000). There are five nephrological centers, including two hospitals. Currently, 172 general practioners ("Allgemeinärzte und Praktische Ärzte"), 123 internists ("Ärzte für Innere Medizin") and 21 urologists are listed in Dortmund. One of the internist practices supervises a dialysis center, but does not provide specialized nephrological consultancy. GPs, internists and urologists were considered primary care providers, as all referrals for treatment of chronic renal failures came from one of these specialties. There were no major fluctuations of physician numbers between 1997 and 1999, the period for which data was analyzed here.

Joint initiatives by all five nephrology centres in Dortmund to give PCPs a series of lectures on treatment of chronic renal disease met with little response, as the presenters regularly outnumbered the audience. In the spirit of the Declaration of Helsinki, part II.1.: ("In the treatment of the sick persons, the doctor must be free to use a new therapeutic measure, if in the doctor's judgment it offers hope of saving life, reestablishing health, or alleviating suffering")[10], K.H. decided to take active measures to induce PCPs to refer patients at an earlier stage.

In order to do so, education was brought to the primary care providers, as opposed to bringing the PCPs to education. Over a period of 18 months, K.H. introduced himself and the potential of timely nephrological care in delaying or even halting the progression of chronic renal disease to PCPs- General Practitioners, and specialists in Internal Medicine and Urology. This introductory round included participation in local PCPs' round tables and PCP organized continuous medical education events, but also individually meeting some 250 PCPs for discussion. During these sessions, the topic of renal disease was subtly introduced during discussions on the more common diseases and risk factors, such as cardiovascular disease, hypertension and diabetes mellitus.

During these teaching sessions and discussions, three arguments were repeatedly brought forward by the PCPs:

1. Mild- moderate renal failure should be treated by the primary care providers, while the nephrologists' task was seen in providing renal replacement therapy.

2. Referral to specialist care meant risking the loss of the patient to the specialist's practice (In Germany, direct specialist access is possible, and patients tend to stay with one doctor if satisfied).

3. If patients did return from specialist consultation, they were usually carrying recommendations for costly permanent medication, such as ACE inhibitors, that put a heavy burden on the PCP's budget.

The first of these arguments was addressed by the "teaching module" of the visits. PCPs were given an executive summary of the complexities of diagnostic and therapeutic procedures used in state- of- the- art nephrology, and the potential of intensified treatment by specialists aiming at slowing the progression of renal failure was explained in detail.

Secondly, all patients referred were asked to report back to the referring PCP within one week of the consultation, by which time a summary of findings and appropriate counsel had been sent ahead.

Thirdly, instead of drug *recommendations*, drug *prescriptions *were handed out directly and so allotted to the much higher nephrologist's budget, thus easing the financial pressure on the primary care providers. More importantly, also the follow- up prescriptions were done in the renal care center, so that at no time the PCPs budget became endangered.

The present study investigates the question whether improving the PCP's knowledge about the potential of timely treatment of chronic renal failure, in combination with addressing their economic concerns, succeeded in encouraging timely referral to specialist care.

## Methods

Retrospective analysis of patients' records. Analysis of patient records was carried out by a final year medical student (TDV). All patients had agreed to have their data used for quality control measures at the time of referral. Specifically informed consent was obtained from all patients still alive at the time of data collection. Two groups were selected by date of first contact with the nephrologist: Group A, third quarter 1997, immediately before the start of the initiative, and Group B, third quarter 1999, six months after the last visit to a primary care provider had taken place. All new patients whose records indicated referral for nephrological specialist treatment were included in the study. Criteria were subnormal creatinine clearance (ECC) or elevated serum creatinine, elevated blood pressure, proteinuria, or erythrocyturia. The patients in each of the two groups were divided into three subgroups according to their renal function: mild/moderate chronic kidney disease (MMCKD), ECC > 60 ml/min/1.73 m^2^, moderate chronic kidney disease (MCKD), 60 ml/min/1.73 m^2 ^> ECC > 20 ml/min/1.73 m^2^, and severe chronic renal disease (SCKD), ECC < 20 ml/min/1.73 m^2^. Due to small proband numbers in the subgroups, the null hypothesis ("no inter- group differences") was tested by the non- parametric, two sided Chi- square test. Survival was estimated by Kaplan- Meier analysis. Statistical analysis was carried out using the SPSS v11.5 package.

## Results

**Figure 1:**
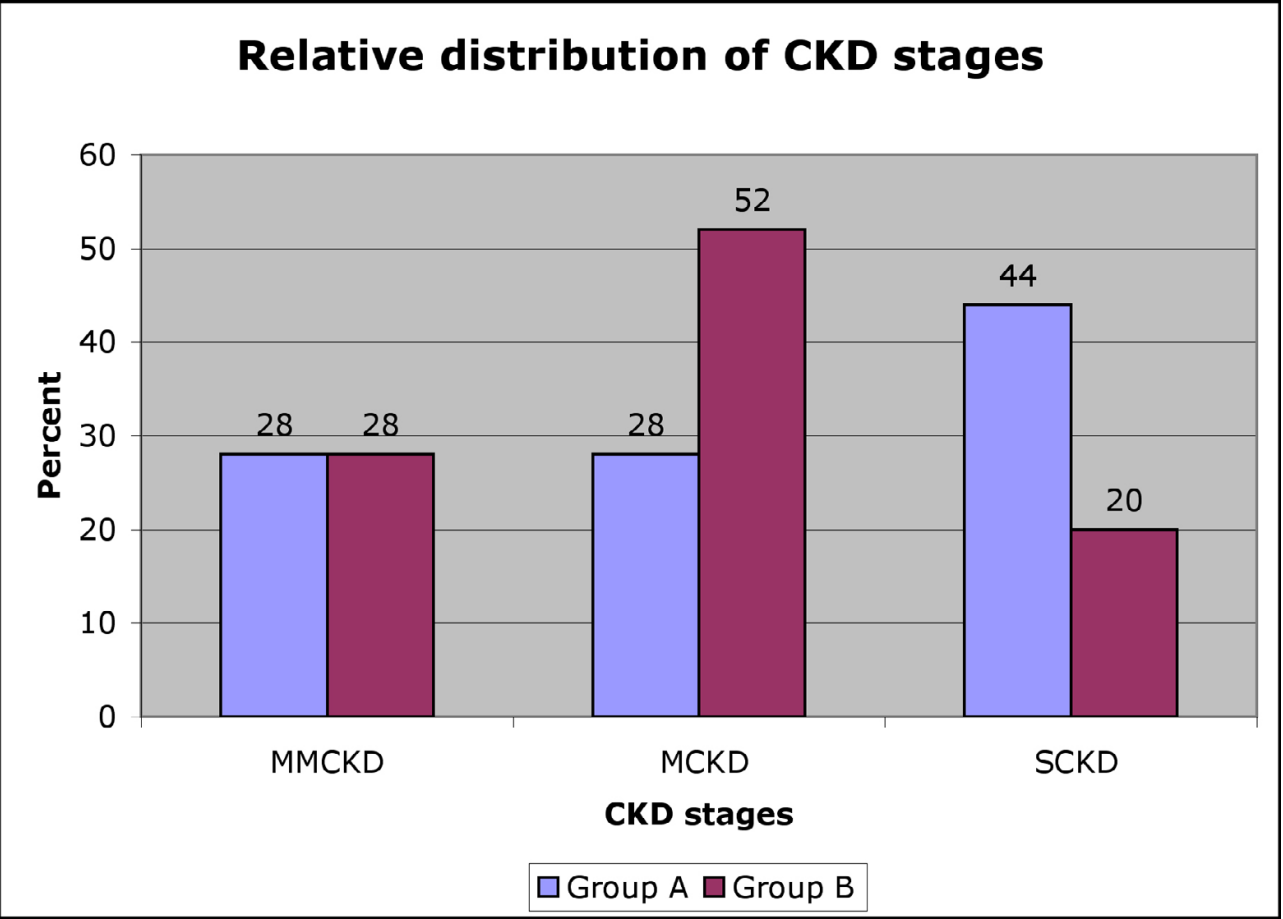
Relative distribution (% of total) of patients into the three subgroups of MMCKD, MCKD and SCKD as defined under "methods". Distribution into MCKD and SCKD is inversed after intervention while MMCKD remains stable. Group A: patients referred in 1997; Group B: patients referred in 1999.

Table 1 and 2 show the descriptive statistics of two groups, including gender and age distribution, diagnosis, prevalence of diabetes, and biopsy frequency. Individually addressing PCPs ignorance and concerns decreased late referral, from the high (SCKD: 44%) to the low (SCKD: 20%) end of the spectrum in published data[5,6] (Chi-square test: 2-sided p = 0.09), as detailed in Figure 1. The relative share of patients seen at the stage of moderate CKD increased, while the relative percentage of patients seen in mild/moderate CKD remained stable. The health outcome (survival in MMCKD and MCKD groups) of patients was insignificantly improved: the mean survival time in group A is 1.71 yrs (1.30–2.12 yrs) compared to a mean survival of 1.90 yrs (1.78–2.02 yrs) in group B.

**Table 1:** Age and gender distribution in the two cohorts.

	**Group A: n= 18**					
	
	**MMCKD**: n= 5 (28%)		**MCKD**: n= 5 (28%)		**SCKD**: n= 8 (44%)	
	
	**M**	**F**	**M**	**F**	**M**	**F**
**Gender (n/ %)**	3/ (60%)	2/ (40%)	4/ (80%)	1/ (20%)	4/ (50%)	4/ (50%)
**Age (yrs ± SD)**	59.7 ± 10.7	44.0 ± 11.3	59.5 ± 20.7	26.0 ± 0.0	55.3 ± 27.2	72.5 ± 8.3
	**Group B: n= 50**					
	**MMCKD**: n= 14 (28%)		**MCKD**: n= 26 (52%)		**SCKD**: n= 10 (20%)	
	**M**	**F**	**M**	**F**	**M**	**F**
**Gender (n/ %)**	11/ (78.6%)	3/ (21.4%)	17/ (65.4%)	9/ (34.6%)	1/ (10.0%)	9/ (90%)
**Age (yrs ± SD)**	32.7 ± 16.1	25.0 ± 17.6	60.9 ± 12.6	54.8 ± 11.8	75.0 ± 0.0	65.4 ± 13.2

**Table 2:** Diagnosis of patients referred for specialist nephrological evaluation.

		**Group A, n = 18 count (% within group)**	**Group B, n = 50 count (% within group)**
**Diagnosis**	No renal failure	0 (0%)	13 (26%)
	Glomerulonephritis	4 (22.2%)	5 (10%)
	Diabetes	3 (16.7%)	8 (16%)
	Nephrosklerosis	2 (11.1%)	4 (8%)
	Lupus	1 (5.6%)	0 (0%)
	Nephrectomy	2 (11.1%)	4 (8%)
	Renal cirrhosis	2 (11.1%)	4 (8%)
	Reflux	0 (.0%)	1 (2%)
	Polycystic disease	1 (5.6%)	1 (2%)
	Others	1 (5.6%)	3 (6%)
	Unknown cause	2 (11.1%)	7 (14%)
**Diabetes**	No	12 (66.7%)	30 (62.5%)
	Yes	6 (33.3%)	18 (37.5%)
**Biopsy**	No	15 (83.3%)	40 (83.3%)
	Yes	3 (16.7%)	8 (16.7%)

## Discussion

The present study is a retrospective analysis of patient data from a single nephrological referral centre, and statistics are carried out on a data set of limited size (n = 18 only in the pre- intervention group A). As such, the statistical significance of the findings is doubtful, and the results might be interpreted as akin to a case report, or a medical anecdote.

As such, however, it may have a value quite different from, but equal to that of a randomized, controlled study.

Aronson recently discussed the value of medical anecdotes[11]; of the eight reasons listed there, the present study meets four: it generates a hypothesis (i.e., close communication with PCPs leads to earlier referral to specialist nephrological care), it suggests a method of management (i.e., individualized teaching sessions and adoption of financial incentives to increase early referral), it reminds and educates (i.e., of the benefit of early referral), and it hopes to stimulate a systematic review (i.e., the authors hope that the results will entice larger dialysis providers to design and carry out a prospective, randomized study).

More often than not, observational studies give similar results to controlled randomized trials[12,13], and critical appraisal of this notion leads to the conclusion that, while good controlled randomized trials do provide the highest level of evidence, a flexible approach may be taken "in which randomised controlled trials and observational studies have complementary roles. High quality observational studies may extend evidence over a wider population and are likely to be dominant in the identification of harms and when randomised controlled trials would be unethical or impractical"[14].

The positive finding of the present study is that active recruiting strategies may improve the referral pattern. Following the discussions with individual PCPs, more patients were referred to specialist care at a stage where appropriate treatment may slow or even halt the progression of chronic renal failure. This should benefit first the patient, and then in the long run society as a whole. Recent studies[2,15] have shown that early referral causes a substantial decrease in hospitalization costs in the first year after referral. It is very probable that adequate treatment and therefore prolongation of the pre- dialysis moderate CKD stage will incur reduced overall spending; the increase in quality of life for the patients is immeasurable. Due to the relatively small number of patients available in this single centre study, combined with the short follow- up interval of just two years, only a slight trend towards prolonged "survival" as defined above could be demonstrated. An American multi- centre study[16] has recently shown, however, that late referral is clearly associated with higher morbidity. To what extend the timing of dialysis initiation influences survival remains as yet questionable. By contrast, Traynor[17] recently demonstrated that early initiation of dialysis may even be detrimental. There is currently one controlled, randomized study addressing this question[18], and the perspective may change once high level evidence is obtained.

Late referral to specialist care in chronic renal disease remains a problem world- wide. As early as 1984, focusing on the question under which circumstances renal replacement therapy should be initiated or not, questionnaires showed significant differences in the attitudes of nephrologists and non- nephrologists towards referral[19] in the United Kingdom, and this difference in behavior has decreased, but not been eradicated over time[20]. Similar observations were made in Canada[1], and the United States [21].

It has been speculated that this may be because these recommendations address the specialist, rather than the primary care providers (PCP)[1,2].

At present, a medline search using the keywords "referral" and "chronic renal failure" listed a total of 81 references since 1974, with only 48 in specialist, not necessarily nephrological journals, while adding "guideline" reduced the reference list to a disappointing three references from the years 1998 and 1999, which included, however, a fully accessible online guideline in the Canadian Medical Association's journal. Neither the American, nor the European Renal Associations' guidelines[3,4] were found by these obvious search strategies. Only the former was found by the search terms "guideline" and "kidney disease", leading to the relevant article in the American Journal of Kidney Disease, accessible only to subscribers.

Better results were obtained using the same search terms on a general search engine (Google), leading at first hit to the DOQI webpage, which includes the free access to full text guidelines. An extensive search using the same terms in German, however, showed no relevant results but an abundance of lay articles of dubious quality. This scarcity of qualified information might exclude those physicians not fluent in English from gathering relevant and up- to- date information. National renal care associations should therefore consider extending their educational efforts beyond their specialist members and selectively target PCPs and their respective professional associations.

In the present study, two arguments brought forward by PCPs to explain their reluctance to refer early focused on the economic burden this presented to their own practice, rather than society as a whole. These two arguments concerned budget penalties if too much money was spent on the (costly) pre- dialytic patients with chronic kidney disease, and loss of patients after referral. This is an unusually frank statement.

The majority of 22 medline hits using the search terms "economy" and "renal disease" address the question of best cost- efficiency of treatment strategies on a *macro*- economic level. *Micro*- economic considerations in the distribution of health care are only hinted at[2]. Health care planners increasingly stress economic factors. While their laudable aim is to provide affordable treatment options to the general population, the drawback of this approach becomes obvious in situations as these, where a conflict of interest arises between best practice and best revenue.

Due to the retrospective design of this study, one cannot analyze to what extent the PCPs' economic considerations rather than their educational state were responsible for their referral pattern. The fact that financial concerns were the tenor of the individual discussions, however, indicates that health care planners may profit from taking this into account.

The authors hope that this single- centre study will entice multi- centre RRT providers to conduct a prospective, large scale study to further investigate the relative contribution of the factors discussed here.

## Conclusions

The initiative presented here shows that timely specialist referral in renal care can be achieved. National and regional differences in the organization of health care provision may lead to variant strategies to implement optimal health care, but close collaboration with the primary care providers is essential.

## List of abbreviations

ECC: endogenous creatinine clearance

MCKD: moderate chronic renal disease

MMCKD: mild/moderate chronic renal disease

PCP: primary care provider

RRT: renal replacement therapy

SCKD: severe chronic renal disease

## Competing interests

The authors declare that they have no competing interests.

## Authors' contributions

K.H. started the initiative, carried out all the teaching modules and gave access to the data.

T.D.V. retrieved the data from archived files

Z.A.L. did statistical analysis

M.L. did statistical analysis

A.M. did statistical analysis and wrote the manuscript.

## Pre-publication history

The pre-publication history for this paper can be accessed here:


